# Understanding Behavioural Determinants of Cardiovascular Disease and Stroke Prevention Among Culturally and Linguistically Diverse Communities: A Qualitative Descriptive Study

**DOI:** 10.5334/gh.1572

**Published:** 2026-07-02

**Authors:** Sabine M. Allida, Della Maneze, Henok Mulugeta Teshome, Scott William, Maree Hackett, Caleb Ferguson

**Affiliations:** 1 School of Nursing, Faculty of Science, Medicine and Health, University of Wollongong, Wollongong, NSW, Australia; 2 Centre for Chronic & Complex Care Research, Blacktown Hospital, Western Sydney Local Health District, Blacktown, NSW, Australia; 3 NHMRC Centre for Research Excellence to Accelerate Innovation and Translation in Stroke Trials, Westmead, NSW, Australia; 4 Therapeutics Initiative Department of Anesthesiology, Pharmacology & Therapeutics, University of British Columbia, Vancouver, Canada; 5 The George Institute for Global Health, Faculty of Medicine and Health, University of New South Wales, Sydney, NSW, Australia; 6 Faculty of Health and Care, University of Central Lancashire, Lancashire, United Kingdom

**Keywords:** CALD, cardiovascular disease, stroke, culture, language, prevention, qualitative

## Abstract

**Background:**

Cardiovascular disease (CVD) and stroke disproportionately affect culturally and linguistically diverse (CALD) communities. In Australia, CALD describes communities that differ from the dominant population by language, ethnicity and/or cultural beliefs and practices. CALD status often intersects with social disadvantage, including lower socioeconomic position, experiences of discrimination and reduced access to culturally safe health services, contributing to inequities in cardiovascular risk and outcomes. Understanding how these factors influence engagement in CVD and stroke prevention activities is essential for developing culturally responsive education and behaviour change programmes.

**Aims:**

This study explored: (i) the experiences of four CALD communities with risk factors for, or history of, CVD or stroke; (ii) their perceptions and prior experiences of CVD and stroke prevention education and (iii) preferences for the content, timing, format and delivery of future prevention programmes.

**Methods:**

Focus groups were conducted face-to-face and via videoconference, audio-recorded and transcribed verbatim. Data were analysed in NVivo using deductive thematic analysis. Themes were mapped to the COM-B model.

**Results:**

Nine focus groups involving 38 participants from Arabic-, Dari-, Chinese- and Vietnamese-speaking communities with risk factors for, or a history of, CVD or stroke were conducted. Nine key themes were identified. Participants described a perceived inevitability of disease, uncertainty about risk factors and the impact of physical limitations on prevention (psychological and physical capability). Engagement was influenced by the need for culturally tailored, accessible education (physical opportunity) and preferences for group-based, community-led support (social opportunity). Motivation and sustained engagement were influenced by cultural food practices, fear of adverse health consequences and inconsistent self-management.

**Conclusion:**

Engagement in CVD and stroke prevention among CALD communities is influenced by social and structural factors beyond individual knowledge or motivation. Findings highlight the importance of culturally tailored, codesigned prevention approaches delivered through trusted community settings and supported by social and healthsystem partnerships to promote more equitable prevention.

## Graphical Abstract

**Figure:**
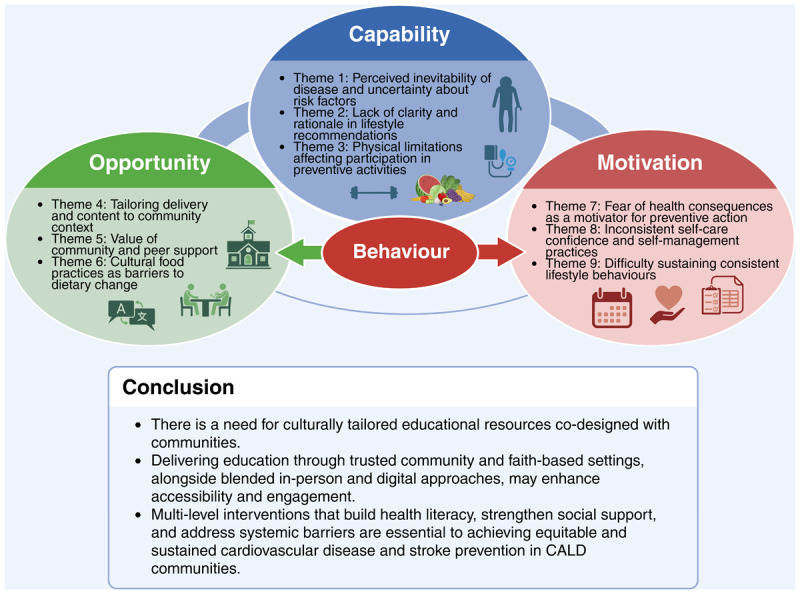


## Introduction

Cardiovascular disease (CVD) and stroke are leading causes of premature death worldwide, significantly contributing to health system burden and reduced quality of life (1). In 2022, CVD accounted for nearly 20 million deaths, comprising about 32% of all deaths globally, most of which (85%) were attributable to heart attack and stroke (1). Similarly, in Australia, an estimated 1.3 million adults (6.7%) live with one or more conditions related to heart disease, stroke and other CVDs. In 2022, CVD was the underlying cause of approximately 45,000 deaths, accounting for 24% of all deaths (2). Prevention is the most effective strategy to reduce CVD incidence and improve long-term outcomes (3). Central to prevention is individual engagement in risk-reducing behaviours such as healthy eating, regular physical activity and exercise, smoking cessation and medication optimisation, adherence and persistence. The success of these strategies also depends on individuals having the capability, opportunity and motivation to undertake and sustain behaviour change.

Australia is a highly multicultural nation with a long history of sustained immigration. Data from the 2021 Census indicate that 28% of the population was born overseas, and approximately one-quarter of Australians spoke a language other than English at home (4). More than 300 languages were reported as spoken in Australian households. Culturally and Linguistically Diverse (CALD) is a commonly used term in Australia to describe individuals who differ from the dominant population by language, ethnicity and/or cultural beliefs and practices (5,6). Earlier policy reviews showed that CALD individuals with limited English proficiency were disproportionately represented among socioeconomically disadvantaged groups (7,8). Recent national data indicate that these disparities persist, particularly in relation to employment outcomes and access to services (9). These structural inequities are reflected in health outcomes, with CALD communities experiencing disproportionately high rates of CVD and stroke, as well as poorer health outcomes compared with the general population (10). Although these disparities are well recognised, there is limited empirical evidence examining how intersecting social and structural factors influence participation in and engagement with CVD and stroke prevention behaviours. As a result, existing prevention strategies may not adequately reach or align with the needs and lived experience of these populations. Understanding how individuals engage with current prevention approaches and the factors influencing this engagement is critical to informing more equitable prevention strategies and reducing persistent inequities in cardiovascular health among migrant and minoritised populations, globally.

This paper reports findings from the second phase of a three-phase study to co-design an educational behaviour change programme for CALD communities. The first phase, reported elsewhere (11), examined healthcare professionals’ and key health networks and non-government organisations’ perspectives on barriers and facilitators to CVD and stroke prevention among CALD communities in Australia. The study identified the influence of social disadvantage, limited health literacy, and culturally mediated beliefs about illness and prevention (11). Building on these findings, this second phase comprised formative engagement with Arabic-, Chinese-, Dari-, and Vietnamese-speaking communities affected by CVD, stroke or associated risk factors, such as hypertension and type 2 diabetes. These communities were selected based on evidence of disproportionately elevated CVD and stroke risk and a high prevalence of modifiable cardiovascular risk factors within these populations in Australia (12). This study aimed to explore their lived experiences of CVD, stroke or associated risk factors, perceptions of existing CVD and stroke prevention education and community preferences for future programme content, timing, format and delivery. These insights were used to inform the subsequent co-design and intervention development and evaluation phase.

## Methods

### Design and theoretical framework

A qualitative descriptive study was conducted using focus group discussions. This study is reported using the Consolidated Criteria for Reporting Qualitative Research (COREQ) checklist (13) (see Supplementary).

**Figure 1:**
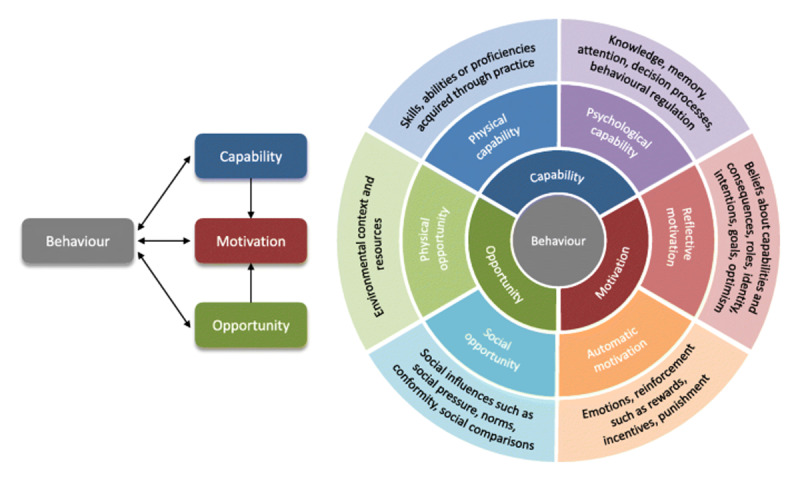
The COM-B model. Reproduced from McDonagh et al 2018 under the terms of the Creative Commons Attribution 4.0 International License.

The COM-B model (Capability, Opportunity and Motivation – Behaviour) (14) was used to develop the interview guide and as the organising framework to explore each community’s knowledge and skills (capability), their environmental resources and societal influences (opportunity), and the reflective and automatic processes that influence motivation. By conceptualising behaviour as the outcome of dynamic interactions between capability, opportunity and motivation (Figure 1), the model enables a comprehensive understanding of the multiple factors influencing engagement with CVD and stroke prevention education, as well as behaviour change. This framework provided a robust theoretical foundation for guiding the next phase of the study, which will co-design a culturally tailored educational behaviour change programme.

### Participants, setting and recruitment

Purposive and snowball sampling were used to recruit eligible adults from Arabic-, Chinese-, Dari-, or Vietnamese-speaking communities who self-reported at least one of the following conditions: hypertension, dyslipidaemia, Type 2 Diabetes Mellitus (T2DM) or a history of CVD e.g., atrial fibrillation or stroke or transient ischaemic attack. Recruitment strategies included distributing flyers through a two-site local health district general hospital and an affiliated community health centre in Western Sydney Local Health District (WSLHD), New South Wales (NSW). WSLHD was selected due to its substantial cultural and linguistic diversity and elevated cardiovascular risk. According to the 2021 Census, over 50% of residents in WSLHD speak a language other than English at home, higher than the NSW average (15,16). This two-site general hospital in WSLHD provides services to catchment areas with high proportions of CALD residents and socioeconomic disadvantage and provides care to communities with a high burden of CVD and related risk factors. They offered practical feasibility through established clinical and community referral pathways. Invitations were also shared via the Stroke Recovery Association NSW (https://strokensw.org.au/) and the Stroke Foundation’s EnableMe (https://enableme.org.au/) community platform, as well as through partnerships with two CALD community organisations—the Vietnamese Community in Australia (Wollongong) (https://www.vcawol.org.au/) and the Chinese Christian Community Service Centre (https://www.acnc.gov.au/charity/charities).

### Ethical considerations

The study was approved by the Western Sydney Local Health District (WSLHD) Human Research Ethics Committee, New South Wales, Australia (ETH01328; 31 July 2023). Study information was provided to all participants, highlighting that participation was voluntary and all identifying details would be removed to ensure anonymity. All participants provided written informed consent. Participants received a $50 grocery gift card as reimbursement for their time and participation.

### Data collection

Between March and September 2024, focus groups were conducted either in person at community centres, libraries or places of worship or remotely via video conference. Sessions were conducted in English or participants’ primary languages by two experienced qualitative researchers (SA and DM). For sessions conducted in other languages, National Accreditation Authority for Translators and Interpreters (NAATI)-accredited interpreters provided real-time interpretation and accurate communication between participants and researchers. Focus groups were recorded and transcribed verbatim by Pacific Transcription. Audio recordings captured participants’ original language and the interpreter’s English translation. To preserve meaning and cultural contexts, the researchers verified key terms, phrases and culturally specific concepts with bilingual participants and the interpreter during the focus group discussions to ensure accurate representation of participants’ intended meaning. Transcripts were produced in English and used as the primary data source for analysis. The interview guide was developed based on existing literature, the COM-B model and the research teams’ experience working with multicultural communities. Refer to Table 1 for the interview guide. Data collection continued until no new concepts were found, and the final sample size was determined based on information power (17). Both researchers maintained field notes and exchanged reflective observations, which were collaboratively used to inform the final themes. A demographics survey, administered via REDCap hosted by the New South Wales (Australia) Ministry of Health, Office of Health and Medical Research (18,19), collected data on participants’ age, sex, country of birth, language spoken at home, educational attainment, years since arrival in Australia, diagnosis of CVD, stroke and/or associated risk factors.

**Table 1:** Interview guide.


INTERVIEW GUIDE	

**1.**	As someone from the Arabic-, Chinese-, Dari-, or Vietnamese-speaking community, what has been your experience of living with high blood pressure, high cholesterol, diabetes, or stroke?

**2.**	What concerns you most about being diagnosed with these conditions?

**3.**	Can you tell me about your family history of high blood pressure, high cholesterol, diabetes, or stroke?

**4.**	What do you think would help you or people from your community better manage these conditions?

**5.**	What do you know about stroke and how it can be prevented?

**6.**	How have you previously received education about stroke?

**7.**	What did you think about the education program? **a.** Was it informative? **b.** Was it easy to understand? **c.** Did you need an interpreter?

**8.**	How do you think you and others in your community can best learn about stroke?

**9.**	In your view, what education opportunities are currently available?

**10.**	What could be improved to better support your community in managing stroke risk and prevention strategies?

**11.**	How would you like to receive information about stroke, your risk, and ways to prevent it? **a.** At what point (e.g., at diagnosis, during hospitalisation) **b.** In what format (e.g., group sessions, one-on-one, digital apps, brochures/pamphlets)?

**12.**	If you were to design a brochure about stroke for your community, what should it include? **a.** Should it be translated?


### Data analysis

Data were analysed using reflexive thematic analysis following Braun and Clarke’s method (20). Analysis was theory-informed and guided by the COM-B model. Initial coding was undertaken deductively using the COM-B domains to guide identification of factors influencing engagement in CVD and stroke prevention. Two authors experienced in qualitative data analysis (SA and HMT) independently read and coded all transcripts using QSR NVivo 15 (21). Codes were reviewed iteratively, with related codes grouped into themes. Themes were grouped under the COM-B domains that most strongly reflected participants’ accounts. Any discrepancies were resolved through discussion with a third author (DM). The COM-B model (14) was used to support the interpretation and organisation of the findings.

### Positionality

SA is a second-generation Filipino Australian cardiovascular researcher, while DM is a first-generation Filipino Australian and an overseas medical doctor with two decades of experience as a multicultural health promotion officer in the local health district. HMT is an early-career cardiovascular researcher from Ethiopia. Although the researchers did not share the same cultural or linguistic backgrounds as the participants in this study, they all have extensive professional experience working with and in CALD communities. This influenced the design and facilitation of the focus groups, including sensitivity to language needs and culturally appropriate engagement. The researchers’ clinical and public health backgrounds may have influenced how participants’ accounts were interpreted, particularly in relation to disease prevention and behaviour change. To support reflexivity, analyses were conducted iteratively within the research team, and interpretations were grounded closely in participants’ reported experiences.

## Results

### Participant characteristics

Nine focus groups were conducted with a total of 38 participants from Arabic-, Chinese-, Dari-, and Vietnamese-speaking communities, with group sizes ranging from two to four participants. Most focus groups were conducted in the participants’ primary languages (Mandarin, Vietnamese and Dari), with only three groups held in English. The average duration of focus groups was 67 (range 49 to 90) minutes.

The participant’s mean age was 66 years, and 75% (n = 29) were women. Most participants (82%, n = 31) reported having one or two CVD or stroke risk factors, and seven participants had lived experience of stroke. Only one had English as the language spoken at home. More demographic details are presented in Table 2.

**Table 2:** General demographic characteristics of participants.


	TOTAL (n = 38)

Mean age (SD)	66 (SD)

Sex, n (%)	

Men	9 (24%)

Women	29 (76%)

Country of birth, n (%)	

Afghanistan	7 (18%)

Australia	1 (3%)

China	10 (26%)

Egypt	8 (21%)

Hong Kong	3 (8%)

Vietnam	8 (21%)

Language spoken at home, n (%)	

Arabic	8 (21%)

Cantonese	3 (8%)

Dari	7 (18%)

English	1 (3%)

Mandarin	10 (26%)

Vietnamese	8 (21%)

Mean years since arrival in Australia (SD)	28 (13.6) years

Level of education, n (%)	

Postgraduate degree	4 (10%)

Bachelor degree	18 (47%)

Diploma or TAFE qualifications	5 (13%)

Secondary School	10 (26%)

Primary School	1 (3%)

Chronic conditions, n (%)*	

Atrial fibrillation	3

Dyslipidaemia	21

Depression	1

Cardiovascular disease	1

Hypertension	24

Kidney disease	1

Myocardial infarction	1

Stroke	7

Type 2 Diabetes	15


* Participants were asked to list current chronic conditions.

### Themes

**Figure 2:**
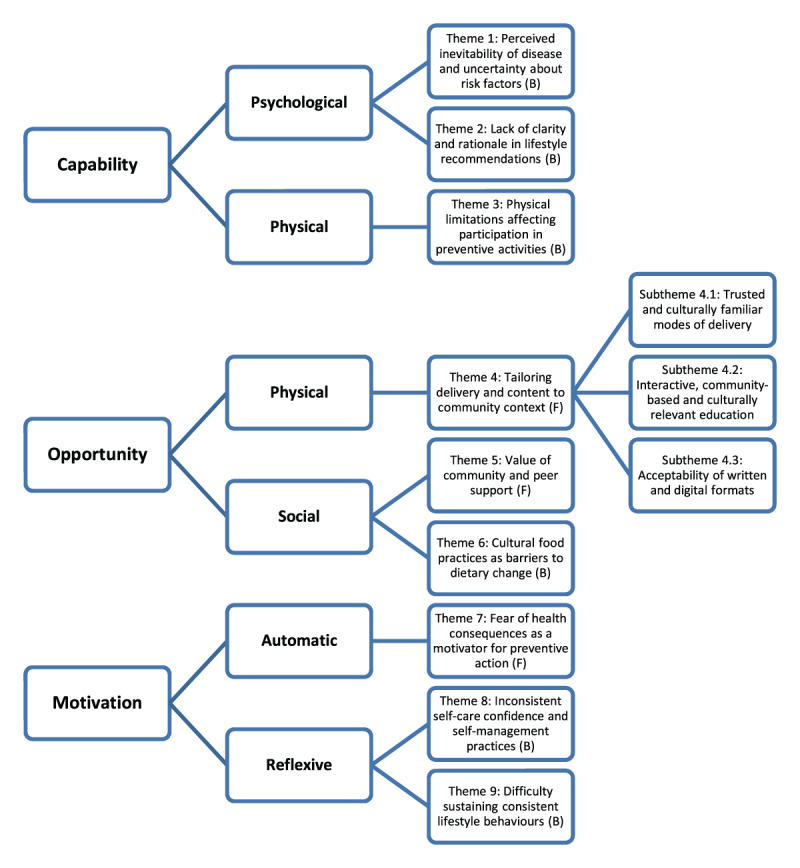
Thematic map.

Themes were grouped under the COM-B domains that most strongly reflected participants’ accounts. (Figure 2).

### Capability

According to the COM-B model, behaviour can only occur when the individual has the necessary capability. This can be ‘psychological’, such as having the knowledge, psychological skills and stamina, or ‘physical’, meaning the individual possesses the physical skills, strength or endurance needed to carry out the behaviour.

### Psychological capability

#### Theme 1: Perceived inevitability of disease and uncertainty about risk factors (Barrier)

In some communities, chronic conditions such as diabetes and hypertension are widely perceived as inevitable, often attributed to ageing or heredity:

‘For [our community], having diabetes is expected. It’s like once you turn 40, 50, you will be diagnosed. But from my point of view, you can prevent it. So, getting them to understand that it’s a preventable disease by managing your weight, managing your diet, I’m finding it challenging’. FG4 – Dari

‘I had prepared to accept this fact because I think it’s hereditary because my grandmother, my mother and my two aunts had hypertension and for my mother, she had hypertension when she’s in 30s’. FG5 – Chinese

Conflicting understanding of risk factors, for example, uncertainty about the role of diet in hypertension, may contribute to scepticism about prevention:

‘I am wondering why many people say high blood pressure is caused by eating too much salt, so why does her husband eat salty [food] and doesn’t have it, while she doesn’t eat salty [food] and got it’. FG7 – Vietnamese

While serious life events like stroke are recognised, their connection to underlying conditions is not always understood:

‘They [the community] know that somebody had a stroke, but I don’t think they really know that hypertension is one of the causes’. FG1 – Arabic

#### Theme 2: Lack of clarity and rationale in lifestyle recommendations (Barrier)

Participants described guidance on exercise and diet as too general and lacking clear rationale. Without specific guidance or visible outcomes, their benefits were difficult to recognise, which decreased motivation and made engagement with lifestyle recommendations more challenging:

‘That’s quite too general to say to exercise. Do exercise is easy to say. So, what kind of exercise, how long for the exercise, how often do I have to do the exercise? You know, walking is exercise, to the gym is an exercise. So, how can we [do it]? We can’t see the good of it, so if you go for exercise, half an hour a day or 10 minutes or 20 minutes a day. We can’t see what’s the benefit of it’. FG2 – Chinese

‘You tell people, don’t eat this. Don’t eat this. Eat this, eat that. But if you tell them why, they’re more prone to comply than when they don’t know why’. FG1 – Arabic

### Physical capability

#### Theme 3: Physical limitations affecting participation in preventive activities (Barrier)

Physical limitations were identified as significant barriers to participation in CVD and stroke-preventive activities. Mobility challenges, chronic pain, and fear of injury restricted opportunities for exercise and attendance at educational sessions, even when individuals expressed interest:

‘I am interested to do some exercises, but I have back pain. I had an injury. I had surgery. I had a bit of – weakness in the left side of my body…and I’m scared for falling’. FG1 – Dari

‘It’s not convenient for me to walk to that lecture because of my legs…’ FG4 – Chinese

### Opportunity

In the COM-B model, there must be conducive physical and social environments for behaviour to occur. ‘Physical’ opportunity refers to environmental factors such as available time, resources and location, while ‘social’ opportunity refers to external social and cultural factors such as norms, social cues and peer support that make a behaviour easier or harder to carry out.

### Physical opportunity

#### Theme 4: Tailoring delivery and content to community context (Facilitator)

##### Subtheme 4.1. Trusted and culturally familiar modes of delivery

Different communities have varied preferences for how they would like to receive CVD and stroke prevention information. Community radio was described as a trusted source, particularly among older Arabic-speaking members, with messages seen as more effective when delivered by health professionals in the community’s language:

‘But she [elderly mother] gets a lot of her news, a lot of the information from the community radio and if you have a doctor on there. Something that they can identify with I think is very important. Especially coming from someone that speaks their language’. FG1 – Arabic

##### Subtheme 4.2. Interactive, community-based and culturally relevant education

Participants across groups expressed a preference for interactive, face-to-face formats. Demonstrations, visual aids, and opportunities for discussion were also viewed as essential for making health information clear, memorable, and relevant:

‘Have a workshop, maybe have a video to demonstrate what is the symptom to let them know much clearer, with video is quite easier…’ FG2 – Chinese

‘It’s good to have an open discussion and that will probably stick in their heads better, that I learned this’. FG4 – Dari

Community spaces, such as mosques and libraries, were identified as suitable venues for prevention education, for some communities:

‘Because a mosque is a place [where] everybody goes regularly. They have that type of stuff for youngsters right now in the mosque. They have like a Saturday night where the sheikh will do a speech and then after that, to encourage the youth, they’ll have a barbecue and then people can sit down and talk to each other. Just to make it a friendly environment for people to gather and talk’. FG4 – Dari

‘After they retire [older generation], they do have time and if we have a quick little library session – have a library meeting room for a little group at a time’. FG3 – Chinese

Participants also found that taking health messages home to share with family was valuable because it reinforced learning and had an impact:

‘I cannot see why not have something [written materials] to take home for the family as well. If you’re going to teach them how to recognise the signs of stroke and to call 000, that might not be a bad idea as well’. FG3 – Chinese

Beyond preferred modes and settings, participants emphasised that the content of prevention education needed to be culturally relevant or else they may be ignored:

‘I feel like in a busy world, working and if that information is not related to me, I may not take the time to investigate and understand it’. FG2 – Chinese

One participant gave an example in the context of existing healthy eating advice. They felt that advice given is mainly focused on common or Western foods and habits:

‘That would be great based on Chinese food. Different culture have different foods. They cook differently but – most of the [healthy eating] advice is based on the local person [meaning, Western]’. FG2 – Chinese

In addition to cultural relevance, participants also stressed the need for information to be presented in simple, clear language to support understanding. Written materials that relied on complex terminology or literal translation were often set aside. Differences in language and dialect further complicated comprehension, with some participants noting that materials could remain difficult to understand even when provided in their primary language:

‘They might pick it up and just read the title – it has to be very interesting and written in very simple language. Because if it’s written in very complicated languages, a lot of terminology, they will look at it and think I will look at that later. They haven’t got time for that. It’s laid aside and then into the bin. In most cases, they just give the text to a translator. They translate it into Arabic and there, it’s translated. But whether everybody understands what’s written, is something else. There’s different types of Arabic. What we might have a word for a cupboard, [in] Lebanese, it’s a tyre in Egyptian’. FG1 – Arabic

Discussion among Arabic participants also stressed the importance of attention-grabbing messages:

‘Participant 1: The name of the video. It was the title. For example, stop, be careful.

Participant 2: When he say, be careful, don’t do that. Be careful. Eat only this five vegetables… Be careful from this one. It will destroy your life.

Participant 1: Unfortunately, maybe it’s wrong, but that’s what gets attention for us. Maybe if you are more honest, it will take our attention…’ FG9 – Arabic

Visual cues, such as the images on cigarette packaging, were also noted as effective for conveying health risks quickly:

‘But like smoking, that horrible package that tell people, hey, look at the pack, the cigarette, you know what the sign, what’s the problem if you take it. That is a quick I get the information without people telling me’. FG2 – Chinese

##### Subtheme 4.3. Acceptability of written and digital formats

Traditional printed materials were increasingly seen as an ineffective strategy:

‘I also don’t believe much now in printing a lot of those booklets. Brochures now, people just take it. You even hand out booklets – it might not reach their homes. After a community group [session], you distribute health education printed material. Okay, if it ends up in their home, thank God for that, but after home, it will be laid aside’. FG1 – Arabic

‘Because a lot of time, if you give papers, they just take it home, and they won’t read it’. FG4 – Dari

Participants described mixed experiences with using digital technology. Some saw value in emails, messages or chats for sharing health information:

‘But the emails perhaps would be a good thing. Or messages or chats’. FG9 – Arabic

However, generational differences may influence accessibility:

‘…older generation, they don’t like the computer and on the phone’. FG8 – Vietnamese

The younger generation was perceived as more comfortable with digital platforms, whereas the older generation often relied on family support to navigate them:

‘For the younger generation, it would work but for the older generation, I don’t know. I’m thinking of my mum. She would probably just hand me the phone and say, just deal with this’. FG1 – Arabic

‘My son helps because we are not good at using this [pointing to the phone]’. FG5 – Chinese

There were also concerns about limited digital literacy and difficulty searching for information:

‘So mobile phone is fine, but sometimes we don’t know how to search’. FG5 – Chinese

‘They might play with their phones okay, but if you tell them to google [the] Heart Foundation, it maybe not be the case’. FG3 – Chinese

Suspicion of online scams also reduced confidence in digital formats:

‘You have to be very careful though nowadays because otherwise they think the message is a scam’. FG3 – Chinese

### Social opportunity

#### Theme 5: Value of community and peer support (Facilitator)

Participants valued community group-based learning for the opportunity to share experiences, exchange ideas and provide peer support:

‘I like group because I can hear [what] other people are saying – everybody have some idea, more or less, we can know about it. So, learn from each other, and support each other. I’m not the only one with the problem’. FG3 – Chinese

Meeting in community groups was described as fostering connection, friendship and a sense of not being alone in facing health challenges:

‘Meeting in community groups, when people get together who speak the same language and have the same background. They sit and they chat – they make connections and friendships together’. FG9 – Arabic

In addition to in-person gatherings, some participants also suggested using digital platforms for administrative purposes to help extend the sense of group support and maintain ongoing reminders, coordinate educational sessions and encourage accountability among peers:

‘Make a group [in WhatsApp], keep reminding each other. Let’s get together for something [education session]’. FG9 – Arabic

‘It’s the lecture for diabetes. On the WeChat – it always organises. So, this club is for eye diseases related to diabetes’. FG4 – Chinese

#### Theme 6: Cultural food practices as barriers to dietary change (Barrier)

Discussions among Vietnamese participants highlighted cultural dietary practices, noting a preference for salty foods and rice as dietary staples. These were seen as central to everyday eating habits but also recognised as potential contributors to health risks when consumed in excess:

‘Participant 1: Most Vietnamese people when they’re still young, they prefer to eat salty.

Participant 2: Dried fish.

Participant 1: And rice, too much rice.

Participant 2: Our traditional [way] is we eat; we cook with rice’. FG8 – Vietnamese

They also reflected on the frequent use of fatty meats and the practice of cooking with rendered fat or oil. These habits were described as traditional but also linked to concerns about elevated cholesterol:

‘Pork belly – we eat a lot of that. And the fat. We’d fry them and we’d cook with the fat of the oil. And we eat the dry one. That’s the reason why we’ve got more cholesterol’. FG8 – Vietnamese

Dietary practices were closely tied to cultural identity, described as central to the preparation and enjoyment of food. Participants felt that reducing ingredients was seen as compromising taste and cultural tradition:

‘But generally speaking, the Arab community is very hard to separate salt from them. Like you said, if you don’t put salt and you don’t put ghee and you don’t put this and you don’t put that, food becomes very bland. And to us, our life is food’. FG1 – Arabic

Food was also described as central to social and cultural gatherings, making dietary change particularly challenging. Celebrations and community events were strongly associated with food preparation and sharing, reinforcing established eating practices:

‘I think the food is the most important – difficult thing to manage for me. Because all the occasions, you know? Generally speaking, Arabs when they get together it’s all about food. You celebrate and the first thing you think, what am I going to make?’ FG1 – Arabic

### Motivation

Motivation is a core component of the COM-B model, emphasising that a behaviour will only occur when there is sufficient motivation. Reflective motivation refers to conscious decision-making, planning and evaluations. In contrast, automatic motivation involves more instinctive drivers, such as desires, needs, impulses and habitual or reflexive responses.

### Automatic motivation

#### Theme 7: Fear of health consequences as a motivator for preventive action (Facilitator)

Fear of serious health consequences served as a strong motivator for adhering to self-management practices as a way of reducing perceived risk or avoiding institutional care or loss of independence:

‘I know if I don’t get it [hypertension] under control then the problem, it’s going to come. I don’t want to have a big stroke and end up in a nursing home’. FG3 – Chinese

‘I have problem with my heart. If today I have no time to walk, and when I check in here [pedometer] that I did around 5,000 steps only. Just before we have dinner, I do some aerobics at home. I just look in the video and I try to do it to complete 10,000 steps because otherwise I’m afraid I’ll die soon’. FG7 – Vietnamese

### Reflective motivation

#### Theme 8: Inconsistent selfcare confidence and selfmanagement practices (Barrier)

There are gaps in self-care confidence and skills within communities. Some participants described taking personal responsibility for monitoring and managing their blood pressure.

‘I’m sometimes alone so, when my blood pressure goes up. I have to know how to treat myself’. FG4 – Dari

‘Little things I do now will prevent a bigger problem down the track. I watch a bit more on my diet’. FG2 – Chinese

While these practices reflected an awareness of prevention and self-management, they were often applied inconsistently or only in response to symptoms, rather than as part of a sustained routine:

‘I had never had a measuring device before, but since it was high [blood pressure], I bought one to measure it because I knew when it went up. But I only measured it if I felt different’. FG8 – Vietnamese

These inconsistent practices were reinforced by a broader reactive orientation to health, in which preventive action was often postponed until problems became unavoidable:

‘The community are not well aware of health issues. Like we’ll cross that bridge when we get there. So, if it’s not affecting them right now, they don’t care about it’. FG9 – Arabic

‘Rather than being proactive, they’ll wait for something to happen which is a problem’. FG4 – Dari

Some participants also expressed reluctance to engage with preventive information, reflecting fears that reading about illness might lead to experiencing it:

‘Participant 1: Pessimists and optimist.

Participant 2: So yeah, if I read about this, I might have it.

Participant 1: Better not to read’. FG9 – Arabic

#### Theme 9: Difficulty sustaining consistent lifestyle behaviours (Barrier)

While participants acknowledged the importance of exercise, they highlighted difficulties in maintaining consistency. General advice to ‘do exercise’ was perceived as ineffective and insufficient, with a need for more practical guidance on how to incorporate physical activity into daily routines in a sustainable way:

‘My GP told me you have to do exercise, I said, I know, how. We all know, but how we can make it consistent’. FG2 – Chinese

Participants also emphasised the challenge of sustaining lifestyle changes in the context of busy lives. Even when motivated and engaged during education sessions, applying the information afterwards was difficult without ongoing reinforcement:

‘Even though I did take notes all the time. When did I go back to those notes and read and read and applied what I learned? Life, it’s so quick and busy. So, you need constant reminders. But because of the pressures of life. And many things you have to do in a day. And then not enough time. You have to prioritise things. So, you leave this till the end. And for, one day you forget, second day it’s not that important. Third day it’s gone’. FG9 – Arabic

## Discussion

This study highlights the complex interactions among psychological, social, and cultural factors that influence the capacity of CALD communities to engage in CVD and stroke preventive behaviours. Findings show that engagement in CVD and stroke prevention is influenced by social determinants of chronic disease and wider health inequities rather than by individual knowledge or motivation alone. Social and structural influences, including health literacy, access to culturally appropriate information, language barriers and the conditions in which preventive behaviours are expected to occur, played a central role in shaping prevention experiences. At a local level, the findings provide context-specific insights into how existing prevention messages and services are experienced by CALD communities and will inform the co-design of a culturally tailored education and behaviour change programme with community and health-service partners. These findings are also consistent with international evidence (22,23), highlighting their relevance beyond this setting and confirming their applicability to other contexts where social disadvantage, migration and structural barriers intersect with chronic disease prevention.

A central barrier identified was uncertainty about CVD and stroke risk and prevention. Participants commonly described chronic conditions such as type 2 diabetes and hypertension as expected or inevitable, often linked with ageing or heredity. These perceptions intersected with uncertainty about how preventive behaviours, particularly the role of diet in hypertension, affect disease risk, consistent with broader literature on the cross-cultural misinterpretation of biomedical health messages (24). It is also important to note that challenges in understanding the role of diet in hypertension are not unique to CALD communities and have also been reported in English-speaking populations across socioeconomic groups (25,26). Given the older profile of participants, these perceptions may also reflect generational differences in exposure to prevention messaging and access to health information. These findings emphasise the need for culturally and linguistically tailored education, as well as approaches that explicitly engage with existing explanatory models of illness.

The preference for culturally responsive and interactive modes of delivery also aligns with previous findings, demonstrating that health education and promotion may be more effective when delivered through trusted community settings and organisations. For example, CVD risk reduction interventions delivered through faith-based settings/leaders have shown promise in African American and migrant communities in the United States (27,28,29,30). Faith-based environments provide social connection, cultural relevance, and a supportive context in which health education and chronic disease management can be integrated with spiritual practices (31). Through these established networks and relationships, they are well-positioned to strengthen community resilience, reduce health inequities, and reach individuals who may be less likely to engage with conventional healthcare services. Similarly, the emphasis on community radio and face-to-face workshops in this study highlights the continued importance of socially embedded communication channels, particularly for older generations with limited digital literacy. Participants’ mixed experiences with digital formats reflect findings from broader research on the digital divide (32). While younger community members may embrace digital technologies, older adults often rely on family members to mediate access. This intergenerational dynamic represents both a challenge and an opportunity. Digital interventions risk excluding older groups, but family-based models that integrate younger ‘digital mediators’ may extend reach and effectiveness (33).

Consistent with findings from our interviews with healthcare professionals, multicultural health networks and non-government CVD and stroke organisations (11), a recurrent theme of this study was the need for culturally tailored CVD and stroke prevention information and advice. This aligns with previous studies demonstrating that culturally tailored programmes can improve disease awareness in CALD communities, promote long-term adherence to lifestyle behaviours and improve health outcomes (34,35,36). For instance, interventions that incorporate culturally specific dietary examples and cooking practices have been shown to reduce weight, haemoglobin A1C and systolic blood pressure among Korean and South Asian communities (37,38). However, the findings highlight a deeper issue where health information and advice are frequently translated word-for-word, producing educational materials that are accurate in language but culturally irrelevant. Participants in this study further noted that differences in dialects and overly technical terminology compounded these barriers. These insights emphasise the importance of co-design approaches, in which communities are actively involved in shaping the language and framing of CVD and stroke prevention educational materials.

The value placed on group learning, peer exchange, and collective reinforcement highlights the importance of social opportunity in prevention. Networks of support and trust are critical determinants of health behaviours (39). Social networks have the potential to influence health behaviours both positively and negatively (40). Findings from a systematic review and meta-analysis demonstrate that group-based education for diabetes self-management fosters not only knowledge gain but also motivation through accountability and shared identity and improved health outcomes (41). Interestingly, participants in this study also suggested extending such support through digital platforms (e.g., WhatsApp, WeChat). This aligns with previous findings that CALD communities use digital platforms for various purposes, including social interaction, information gathering, everyday routines and leisure (42). However, the generational differences observed here caution against assuming universal digital uptake and highlight the need for blended models that combine in-person and digital forms of support.

The findings also demonstrate that dietary habits, cultural identity and social traditions significantly influence engagement with prevention initiatives. Similar patterns have been reported in studies of Asian, African and Middle Eastern populations, where food practices are strongly tied to cultural identity and social cohesion (43,44,45). Efforts to modify these practices are often perceived as threatening cultural continuity (46), which may explain resistance to dietary advice that advocates reducing staple foods or traditional preparation methods.

Our study also found that some participants tended to delay engaging in prevention until illness became unavoidable, showing a more reactive than proactive approach to health. Similar patterns have been reported in other studies, often linked to competing life priorities, financial pressures, and cultural norms that emphasise immediate concerns over future risks (47). This behaviour does not reflect lack of concern but rather highlights the need for prevention strategies that feel tangible, timely, and personally relevant.

Motivational factors in this study showed two sides: fear of serious consequences, such as stroke or loss of independence, often motivated people to take preventive action, but competing demands and a lack of continued support made maintaining CVD and stroke preventive behaviours difficult. This pattern reflects broader behaviour change research, showing that fear can prompt short-term action (48), but lasting change requires confidence and supportive structures (49). This highlights the need for multi-level interventions that provide not only information but also social, environmental, and health system-level supports to sustain change.

While findings are presented across four CALD communities, several themes were evident across multiple groups, including beliefs about the inevitability of disease, preferences for interactive and community-based education and challenges that sustain preventive behaviours. Concurrently, some themes were more common within particular communities, such as the use of traditional media among Arabic-speaking participants, WeChat-based coordination among Chinese-speaking participants, and culturally specific dietary practices described by Vietnamese and Arabic-speaking participants. These suggest that although key behavioural determinants of prevention are shared, their importance and preferred delivery approaches vary across communities. Future programme development, including the co-design phase of this study, should incorporate common core components with community-specific tailoring.

### Strengths and limitations

Findings are based on a modest, non-representative sample of four CALD communities, which limits the generalisability of the results. However, the inclusion of these communities provided a breadth of cultural perspectives. Another limitation of this study is that, although Arabic-speaking participants were included, all were of Egyptian background, with the majority identifying as Christian and one as Muslim. Consequently, the findings may not fully represent the views and experiences of Arabic-speaking communities from other nations or religious contexts. Although measures were taken to ensure accurate translation and interpretation, it is possible that some cultural nuances may have been lost. However, conducting some focus groups in participants’ preferred languages enhanced inclusivity and contributed to the richness of the data. The sample comprised a greater proportion of women than men, which may have introduced sex and gender-related bias. Health beliefs and behaviours can vary by sex and gender, potentially leading to an underrepresentation of men’s perspectives. In addition, the study did not assess participants’ health literacy, which limits the ability to interpret how literacy levels may have influenced understandings of CVD and stroke prevention. Lastly, member checking could not be undertaken due to limited resources for translation and interpretation. This may have limited the opportunities to validate the accuracy and cultural context of the findings with participants. Importantly, while the findings offer valuable insights, they should be interpreted carefully to avoid overgeneralising or stereotyping cultural groups; individuals within any cultural community hold diverse views and experiences. Nonetheless, the consistency of themes across the four CALD communities and their alignment with existing evidence support the reliability and transferability of the interpretations.

## Conclusion

Complex social, cultural, and psychological factors influencing engagement with CVD and stroke prevention among CALD communities. Perceived inevitability of disease and uncertainty about risk factors and lack of culturally relevant information and advice hindered engagement in preventive behaviours, while social networks and cultural food practices played dual roles as barriers and enablers. The findings highlight that literal translation of CVD and stroke prevention educational materials is inadequate, emphasising the need for culturally tailored educational resources co-designed with communities. Delivering education through trusted community and faith-based settings, alongside blended in-person and digital approaches, may enhance accessibility and engagement. Multi-level interventions that build health literacy, strengthen social support, and address systemic barriers are essential to achieving equitable and sustained cardiovascular and stroke prevention in CALD communities.

## Data Availability

The data that support the findings of this study are available on request from the corresponding author. The data are not publicly available due to privacy or ethical restrictions.
